# Australians support for policy initiatives addressing unhealthy diet: a population-based study

**DOI:** 10.1093/heapro/daad036

**Published:** 2023-05-22

**Authors:** Adyya Gupta, Kim D Raine, Paula Moynihan, Marco A Peres

**Affiliations:** Global Centre for Preventive Health and Nutrition (GLOBE), Institute for Health Transformation, Deakin University, Geelong, VIC 3220, Australia; School of Public Health, University of Alberta, 3-061 Edmonton Clinic Health Academy, Edmonton, AB T6G 1C9, Canada; Adelaide Dental School, Faculty of Health and Medical Sciences, The University of Adelaide, Adelaide, Australia; National Dental Research Institute Singapore, National Dental Centre Singapore, Singapore; Oral Health ACP, Health Services and Systems Research Programme, Duke-NUS Medical School, Singapore

**Keywords:** health policy, public health, public opinion, sugar tax, diet

## Abstract

To inform public health policy implementation in Australia, our study investigated the level of public support for six policy initiatives addressing unhealthy diet. The policy initiatives included taxing soft drinks and energy drinks, taxing less healthy food and beverage purchases, zoning to restrict the supply of junk foods near schools, prohibiting advertising and promotion of less healthy food and beverages to children under the age of 16 and restricting sugar-sweetened beverages from vending machines in schools, and public places. Data from a cross-sectional population-based study for 4040 Australians aged 15+ years, were analysed. A high overall support across all policy initiatives was observed. Nearly three-quarter of public support was observed for policy initiatives targeting children (zoning to restrict the supply of junk food near schools, prohibiting advertising and promotion of less healthy food and beverages to children under the age of 16 and restricting sugars-sweetened beverages from vending machines in schools), and half of Australians supported policy initiatives of taxing soft drinks and energy drinks and taxing less healthy food and beverage purchases. Australian women and those with tertiary level of education were more likely to support public health initiatives targeting children and all policy initiatives respectively. Interestingly, young adults expressed low level of support for all policy initiatives. The study demonstrated considerable public support for policy initiatives focussed on protecting children from unhealthy diet in Australia. Framing, designing and implementing policies targeting children is potentially a good starting point for policymakers to create a health promoting food environment.

## Teaser

Public health policy initiatives addressing unhealthy diet is implemented in many countries with promising results. In Australia, no national-level data are currently available on the public’s opinions towards policy-level strategies addressing unhealthy diet, and whether public opinion varies by demographic characteristics, as seen in other countries. Our findings suggest that framing policies in ways that are focussed on protecting children from unhealthy diet are more likely to gather greater support. Overall, public health advocates and policymakers should emphasise on benefits of implementing population-level initiatives in future advocacy efforts to increase public acceptability for policies to address unhealthy diet.

## BACKGROUND

Chronic non-communicable diseases (NCDs) leads to morbidity and mortality worldwide, accounting for 70% of all deaths (World Health Organization, 2013). An unhealthy diet, high in free sugars, added salt and high saturated fat is a leading but preventable risk factor of NCDs (World Health Organization, 2013). About one-third of the Australia’s dietary energy is sourced from discretionary foods (Australian Bureau of Statistics, 2016a) and over 50% of the Australian population exceeds the World Health Organization’s (WHO) proposed thresholds for free sugars consumption (Gupta *et al.*, 2018b). For the purpose of this study, we used the WHO definition of free sugars, that is, ‘monosaccharides and disaccharides added to foods and beverages by the manufacturer, cook or consumer, and sugars naturally present in honey, syrups, fruit juices and fruit juice concentrates’ (World Health Organization, 2003). Our work established that population-based strategies are necessary to address the societal challenge posed by an unhealthy diet (Gupta *et al.*, 2018a,b, 2019). Moreover, strategies need to deliver equitable benefits given that certain population sub-groups (e.g. Indigenous Australians, low-income families, regional and remote Australians) are at an increased risk of consuming an unhealthy diet. Evidence shows that efforts to address unhealthy diet at the individual level have limited success (Gupta *et al.*, 2018b) and population-level initiatives are needed to achieve population-level improvements in health outcomes.

Creating a healthy environment through population-level initiatives is identified as a priority by the WHO to reduce the burden of non-communicable diseases (World Health Organization, 2022). Evidence on the effectiveness of strategies to address unhealthy diet is well established in different contexts. These include fiscal policy, food reformulation, regulating the volume and content of less healthy food advertising and introducing labelling schemes (such as front-of-pack warning labels, traffic light labelling and other forms of labelling), zoning, subsiding fruits and vegetables and encouraging healthy choices for population-level impact (Mozaffarian *et al.*, 2018; Gupta *et al.*, 2019). Over 50 jurisdictions around the world have implemented sugar-sweetened beverage (SSB) taxes (Global Food Research Program, 2020), and a number of studies have evaluated the effectiveness of the policies (Colchero *et al.*, 2016; Backholer *et al.*, 2017; Silver *et al.*, 2017). Despite the growing body of evidence on the positive health impact of public health strategies there is yet no political consensus on any of these policy-level strategies addressing unhealthy diet in Australia.

Data summarising public support for initiatives addressing unhealthy diet, beyond SSB taxation, are limited and vary within and between countries, policies, and by the sociodemographic characteristics of the population. Public support for an SSB tax in the USA is up to 50% (Rivard *et al.*, 2012; Gollust *et al.,* 2014; Donaldson *et al.*, 2015). Globally, mostly standalone polices (e.g. sugar tax) have received a lower level of public support (Bélanger-Gravel *et al.*, 2019) when compared to a combination of multiple initiatives including text and graphic warning labels on vending machines, SSB advertisement and SSB containers and government-funded TV campaigns about the health effects of SSBs, focussed on children (Kwon *et al.*, 2019; Miller *et al.*, 2019). A lack of national-level evidence of public support is a key barrier for governments to implement policies. In Australia, no national-level data are currently available on the public’s opinions towards policy-level strategies to address unhealthy diet, and whether public opinion varies by socio-demographic characteristics, as seen in other countries. Thus, study aimed to investigate the level of public support towards multiple public health policy initiatives addressing unhealthy diet in Australia and to describe variations in the level of support by socio-demographic factors.

## METHOD

### Data and study population

Population-based cross-sectional data from the 2017 to 2018 National Study of Adult Oral Health (NSAOH) (Peres and Brennan, 2020) were used for this study. The purpose of the NSAOH was to collect data on the oral health status of Australians, and the types and sources of dental care received. The information was collected to inform development of oral health policies in Australia. NSAOH comprised two components: National Dental Telephone Interview Survey (NDTIS) (conducted through Computer Assisted Telephone Interview or online survey) and an Oral epidemiological examination among the dentate population interviewed. NSAOH involved interviews with over 15 000 people aged 15+ years, clinical oral epidemiological examinations of over 5000 people, and the administration of a supplementary questionnaire to those that were interviewed (*n* = 15 000) (ethics approval no. H-2016-046). Based on the scientific evidence on the effectiveness of the policies influencing healthier food consumption (Kongats *et al.*, 2019; Nykiforuk *et al.*, 2019), their relevance in the Australian context (Sacks G. for the Food-EPI Australia project team, 2017; Gupta *et al.*, 2019) and to expand our scientific knowledge regarding what drives public support across more to less intrusive interventions based on their relevance to improve oral health, the NSAOH supplementary questionnaire was adapted from the Canadian Chronic Disease Prevention Survey (PLACE Research Lab, 2015) and included six questions to gather public support for policy initiatives addressing unhealthy diet.

The supplementary questionnaire was self-completed either through a web link or as a paper version. No qualitative data were collected as part of the data used in this study. More details on the survey methodology are presented in the Australia’s Oral Health: National Study of Adult Oral Health 2017–18 report (Chrisopoulos *et al.*, 2019).

### Measures

#### Public support for public health policy initiatives addressing unhealthy diet

Participants responded to the following questions.

1. Do you support taxing soft drinks and energy drinks?
2. Do you support zoning to restrict the supply of junk foods near schools?
3. Do you support taxing less healthy food and beverage purchases?
4. Do you support prohibiting advertising and promotion of less healthy food and beverages to children under the age of 16?
5. Do you support restricting sugar-sweetened drinks from vending machines in all public places?
6. Do you support restricting sugar-sweetened drinks from vending machines in schools?

Proportion of public support for policy initiatives was assessed on a five-point Likert scale (‘strongly agree’, ‘somewhat agree’, ‘neutral’, ‘somewhat oppose’, or ‘strongly oppose’). For meaningful inferences on overall support and overall oppose, we combined the responses for ‘strongly agree’, ‘somewhat agree’ to ‘overall support’ and ‘somewhat oppose’, or ‘strongly oppose’ to ‘overall oppose’.

#### Socio-demographic data

Socio-demographic variables included age group (18–24, 25–44, 45–64 and 65 years and above); sex; highest level of education attained (tertiary bachelor/graduate or post graduate degree), vocational (certificate/diploma), student (studying at a university or school), secondary (no post-secondary qualification and not currently studying); number of members in a household [households with children (0–14 years), households with no children (>15 years)]; total annual household income before tax classified into three categories ($100 000 and above, $50 000 to <$100 000, <$20 000 to $50 000); the index for relative socioeconomic advantage and disadvantage (IRSAD) (Australian Bureau of Statistics, 2016b) was derived from residential postcode and categorised into three categories [most disadvantaged (range 1–5th), medium (range 6th–8th) and most advantaged (range 9th–10th)] and; State or Territory of residence.

### Statistical data analysis

Descriptive statistics were performed on STATA/SE 17.0. To make population-level inferences, all the estimates were weighted to the Australian population [for further details on survey weighting procedures see Ellershaw *et al.* (2020)]. The proportion and 95% confidence intervals for each policy response were estimated by socio-demographic group. This is a secondary data analysis of the subset of the NASOH data and is available on request. The authors had access to only the de-identified data and hence the confidentially of the data was ensured. The study received ethics clearance from the University of Adelaide Research Ethics Committee (H-2016-046/ H-2016-182) and an exemption from the Deakin University Research Ethics Committee (DUHREC#2023-065).

## RESULTS

Out of 15 000 interviewed participants, 4040 responded to the supplementary questionnaire (27%). Nearly one-third of the sample was aged 45–64 years, one-fourth was between 25 and 44 years of age, and less than 10% aged 24 years or less. Females accounted for 54% of the sample; 31% of the sampled household income was $100K and above, and 28% had tertiary education. Seventy-nine percent of households included no children (0–14 years) and 59% resided in medium to most socioeconomic advantaged areas (Table 1).

**Table 1:** Socio-demographic characteristics of the sample (weighted)

Socio-demographic characteristics sample size (*n*)	Proportion of participants (weighted) *n* (%)
Total sample (*n* = 4040)	
Age (*n* = 3986) in years	
18–24	191 (7%)
25–44	1069 (26%)
45–64	1282 (37%)
65 and above	1444 (30%)
Sex (*n* = 4040)	
Male	1696 (46%)
Female	2344 (54%)
Highest level of education attained (*n* = 3983)	
Tertiary	1571 (28%)
Vocational	1577 (47%)
Student	147 (6%)
Secondary	688 (19%)
Number of dependants (*n* = 4034)	
Households with children (0–14 years)	810 (21%)
Households with no children (>15 years)	3224 (79%)
Household income (*n* = 3396)	
$100K and above	1042 (31%)
50K to <100K	881 (25%)
<20K to <50K	1473 (44%)
Area of residence (*n* = 4040)	
Most disadvantaged^*^	1623 (41%)
Medium^**^	1095 (30%)
Most advantaged^***^	1322 (29%)
State of residence (*n* = 4040)^^^	
NSW	607 (20%)
VIC	731 (28%)
QLD	479 (18%)
SA	623 (13%)
WA	465 (15%)
TAS	534 (4%)
ACT	408 (3%)
NT	193 (1%)

^*^Most disadvantaged = range 1–5.

^**^Medium = range 6–8

^***^Most advantaged= range 9–10.

^^^NSW (New South Wales); VIC (Victoria); QLD (Queensland); SA (South Australia); WA (Western Australia); TAS (Tasmania); ACT (Australian Capital Territory); NT (Northern Territory).

Tables 2–7 show the public support level for different policy initiatives by socio-demographic characteristics. Overall, the public support for all policy initiatives addressing unhealthy diet in Australia was high (>55%), with some variability between policies. For policy initiatives targeting children, 77% public support was observed for policy restricting sugar-sweetened drinks from vending machines in schools, followed by 73% public support for policy prohibiting advertising and promotion of less healthy food and beverages to children and 62% public support for policy for zoning to restrict the supply of junk food near schools. For other policy initiatives including taxation of less healthy food and beverage purchases, a relatively lower level (53%) of public support was observed. Socio-demographic variations were observed in the level of support for policy initiatives.

**Table 2:** Crosstabulation between demographic characteristics and support for taxing soft drinks and energy drinks (weighted)^^^^

Demographic characteristics	Support for taxing soft drinks and energy drinks policy		
	Total support^~^ *n*	Neutral^@^ *n*	Total oppose^^^ *n*
Total sample (*n* = 3791)	2296	855	640
	% [95% confidence interval]		
	55.7 [53.4, 57.9]	24.9 [23.1, 27.1]	19.4 [17.7, 21.4]
Age (*n* = 4040) in years			
18–24	45.9 [35.5, 56.5]	36.3 [26.3, 47.1]	18.1 [11.4, 27.6]
25–44	56.3 [51.8, 60.6]	21.9 [18.4, 25.9]	21.8 [18.2, 25.9]
45–64	57.2 [53.3, 61.1]	22.2 [19.0, 25.7]	20.6 [17.6, 23.9]
65 and above	57.0 [53.6, 60.4]	27.2 [24.2, 30.4]	15.8 [13.4, 18.5]
Sex (*n* = 4040)			
Male	55.5 [52.0, 58.8]	23.5 [20.6, 26.7]	21.0 [18.4, 23.9]
Female	55.9 [52.9, 58.8]	26.0 [23.5, 28.8]	18.1 [15.8, 20.7]
Highest level of education attained (*n* = 3983)			
Tertiary	68.5 [65.0, 71.8]	17.9 [15.2, 21.0]	13.6 [11.3, 16.3]
Vocational	53.0 [49.5, 56.4]	25.7 [22.7, 29.0]	21.3 [18.6, 24.4]
Student	52.7 [40.7, 64.5]	27.1 [17.8, 39.0]	20.2 [12.2, 31.5]
Secondary	46.3 [41.3, 51.4]	31.0 [26.5, 35.8]	22.7 [18.7, 27.4]
Number of dependants (*n* = 4034)			
Households with children (0–14 years)	50.4 [45.2, 55.5]	27.7 [23.2, 32.7]	21.9 [17.9, 26.6]
Households with no children (>15 years)	57.2 [54.7, 59.7]	24.2 [22.1, 26.4]	18.6 [16.7, 20.7]
Household income (*n* = 3396)			
$100K and above	63.7 [59.0, 68.1]	18.4 [14.8, 22.7]	17.9 [14.6, 21.8]
50K to <100K	57.0 [52.3, 61.5]	23.9 [20.0, 28.3]	19.1 [15.8, 23.0]
<20K to <50K	54.2 [50.6, 57.8]	27.0 [24.0, 30.3]	18.8 [16.0, 21.9]
Area of residence (*n* = 4040)			
Most disadvantaged^*^	52.7 [49.2, 56.1]	27.1 [24.1, 30.3]	20.2 [17.5, 23.3]
Medium^**^	53.4 [49.2, 57.6]	24.9 [21.2, 28.9]	21.7 [18.4, 25.5]
Most advantaged^***^	62.5 [58.4, 66.4]	21.6 [18.3, 25.4]	15.9 [13.0, 19.2]
State of residence (*n* = 4040)			
NSW	51.0 [45.6, 56.4]	26.6 [22.1, 31.7]	22.4 [18.1, 27.4]
VIC	56.6 [51.9, 61.3]	23.2 [19.3, 27.6]	20.2 [16.6, 24.3]
QLD	53.8 [48.4, 59.1]	24.8 [20.5, 29.9]	21.3 [17.2, 26.1]
SA	51.9 [46.6, 57.1]	29.9 [25.1, 35.2]	18.2 [14.5, 22.7]
WA	62.0 [56.0, 67.7]	22.6 [17.7, 28.3]	15.4 [11.6, 20.2]
TAS	61.5 [56.5, 66.3]	25.6 [21.4, 30.3]	12.9 [9.8, 16.7]
ACT	66.1 [60.2, 71.6]	17.6 [13.7, 22.4]	16.2 [12.0, 21.6]
NT	57.2 [47.7, 66.3]	20.3 [14.1, 28.4]	22.5 [14.9, 32.3]

^*^Most disadvantaged = range 1–5.

^**^Medium range = range 6–8.

^***^Most advantaged = range 9–10.

^^^^Row % estimated presented.

^~^Total support = Strongly support + Somewhat support = Proportion in favour of the policy.

^@^Neutral = Proportion neither for nor against the policy.

^^^Total oppose = Strongly oppose + Somewhat oppose = Proportion against the policy.

**Table 3:** Crosstabulation between demographic characteristics and support for zoning to restrict supply of ‘junk’^#^ food near school (weighted)^^^^

Demographic characteristics	Support for zoning to restrict supply of ‘junk’ food near school		
	Total support^~^ *n*	Neutral^@^ *n*	Total oppose^^^ *n*
Total sample (*n* = 3780)	2446	791	543
	% [95% confidence interval]		
	61.8 [59.5, 64.1]	22.0 [20.2, 24.1]	16.2 [14.5, 18.1]
Age (*n* = 4040) in years			
18–24	43.7 [33.7, 54.3]	36.2 [26.4, 47.2]	20.1 [12.5, 30.6]
25–44	59.2 [54.8, 63.4]	21.0 [17.7, 24.8]	19.8 [16.4, 23.7]
45–64	63.5 [59.7, 67.1]	20.9 [17.9, 24.2]	15.6 [13.1, 18.6]
65 and above	67.4 [64.1, 70.5]	20.6 [18.0, 23.5]	12.0 [9.9, 14.5]
Sex (*n* = 4040)			
Male	61.7 [58.2, 64.9]	22.2 [19.4, 25.3]	16.1 [13.8, 18.8]
Female	61.9 [58.9, 64.7]	21.9 [19.5, 24.4]	16.2 [14.0, 18.7]
Highest level of education attained (*n* = 3983)			
Tertiary	69.0 [65.5, 72.2]	16.4 [13.8, 19.4]	14.6 [12.2, 17.3]
Vocational	62.3 [59.3, 66.0]	22.3 [19.5, 25.4]	15.0 [12.7, 17.7]
Student	45.4 [33.8, 57.6]	31.6 [21.7, 43.4]	23.0 [13.9, 35.6]
Secondary	55.5 [50.3, 60.5]	25.9 [21.8, 30.4]	18.6 [14.7, 23.3]
Number of dependants (*n* = 4034)			
Households with children (0–14 years)	56.9 [51.7, 62.0]	24.6 [20.4, 29.3]	18.5 [14.7, 23.1]
Households with no children (>15 years)	63.0 [60.6, 65.4]	21.5 [19.5, 23.6]	15.5 [13.7, 17.5]
Household income (*n*=3396)			
$100K and above	62.4 [57.8, 66.7]	19.1 [15.7, 23.2]	18.5 [15.2, 22.3]
50K to <100K	60.7 [56.0, 65.2]	23.7 [19.8, 28.0]	15.6 [12.5, 19.3]
<20K to <50K	64.5 [60.9, 67.9]	22.6 [19.7, 25.7]	12.9 [10.5, 15.8]
Area of residence (*n* = 4040)			
Most disadvantaged^*^	62.6 [59.1, 65.8]	23.5 [20.7, 26.6]	13.9 [11.7, 16.5]
Medium^**^	60.9 [56.6, 65.0]	19.2 [16.1, 22.7]	19.9 [16.6, 23.8]
Most advantaged^***^	61.7 [57.5, 65.6]	22.9 [19.5, 26.8]	15.4 [12.6, 18.7]
State of residence (*n* = 4040)			
NSW	59.3 [53.9, 64.5]	24.2 [19.8, 29.1]	16.5 [12.9, 21.0]
VIC	61.8 [57.1, 66.3]	20.2 [16.7, 24.4]	17.9 [14.6, 21.9]
QLD	63.7 [58.3, 68.8]	19.6 [15.7, 24.3]	16.7 [12.8, 21.5]
SA	56.0 [50.6, 61.2]	27.1 [22.6, 32.0]	16.9 [13.1, 21.7]
WA	65.7 [59.7, 71.3]	21.2 [16.6, 26.7]	13.1 [9.3, 18.0]
TAS	65.3 [60.4, 70.0]	23.3 [19.2, 27.8]	11.5 [8.5, 15.1]
ACT	67.5 [61.4, 72.8]	19.5 [15.1, 24.8]	13.0 [9.4, 17.9]
NT	61.5 [51.7, 70.4]	20.5 [14.0, 29.2]	18.0 [11.1, 27.7]

^*^Most disadvantaged = range 1–5.

^**^Medium range = range 6–8.

^***^Most advantaged= range 9–10.

^^^^Row % estimated presented.

^~^Total support = Strongly support + Somewhat support = Proportion in favour of the policy.

^@^Neutral = Proportion neither for nor against the policy.

^^^Total oppose = Strongly oppose + Somewhat oppose = Proportion against the policy.

^#^Junk food refers to discretionary food and drinks high in sugar, salt and saturated fats.

**Table 4:** Crosstabulation between demographic characteristics and support for taxing less healthy^#^ food and beverage purchase (weighted)^^^^

Demographic characteristics	Support for taxing less healthy food and beverage purchase		
	Total support^~^ *n*	Neutral^@^ *n*	Total oppose^^^ *n*
Total sample (*n* = 3791)	2182	842	757
	% [95% confidence interval]		
	53.0 [50.8, 55.2]	24.4 [22.5, 26.4]	22.6 [21.7, 24.5]
Age (*n* = 4040) in years			
18–24	35.3 [25.9, 45.9]	39.0 [29.0, 50.0]	25.7 [17.1, 35.7]
25–44	54.5 [50.1, 58.9]	21.1 [17.6, 25.1]	24.4 [20.7, 28.4]
45–64	54.2 [50.3, 59.1]	22.4 [19.2, 25.8]	23.4 [20.2, 26.9]
65 and above	55.3 [51.9, 58.7]	26.4 [23.5, 29.5]	18.3 [15.6, 21.3]
Sex (*n* = 4040)			
Male	53.2 [49.8, 56.6]	23.9 [21.0, 27.1]	22.9 [20.6, 25.9]
Female	52.9 [49.9, 55.8]	24.9 [22.4, 27.5]	22.2 [19.8, 25.0]
Highest level of education attained (*n* = 3983)			
Tertiary	64.8 [61.2, 68.2]	18.8 [16.0, 21.9]	16.4 [13.4, 19.3]
Vocational	50.3 [46.9, 53.7]	25.4 [22.4, 28.6]	24.3 [21.4, 27.4]
Student	47.5 [35.6, 59.6]	28.4 [18.9, 40.2]	24.1 [15.4, 35.8]
Secondary	45.3 [40.3, 50.4]	28.88 [24.63, 33.54]	25.81 [21.46, 30.69]
Number of dependants (*n* = 4034)			
Households with children (0–14 years)	48.7 [43.6, 53.9]	27.2 [22.7, 32.3]	24.1 [20.0, 28.8]
Households with no children (>15 years)	54.2 [51.7, 56.7]	23.8 [21.7, 26.0]	22.0 [19.9, 24.2]
Household income (*n* = 3396)			
$100K and above	59.4 [54.8, 63.9]	18.1 [14.6, 22.2]	22.5 [18.9, 26.6]
50K to <100K	52.9 [48.2, 57.6]	26.2 [22.0, 30.8]	20.9 [17.5, 24.7]
<20K to <50K	54.1 [50.5, 57.7]	26.0 [23.0, 29.3]	19.9 [17.1, 23.0]
Area of residence (*n* = 4040)			
Most disadvantaged^*^	51.8 [48.3, 55.2]	24.9 [22.1, 28.1]	23.3 [20.4, 26.5]
Medium^**^	50.7 [46.4, 54.8]	23.7 [20.1, 27.5]	25.6 [22.1, 29.7]
Most advantaged^***^	57.4 [53.3, 61.4]	24.5 [21.1, 28.4]	18.1 [15.1, 21.5]
State of residence (*n* = 4040)			
NSW	47.5 [42.4, 53.13]	27.2 [22.5, 32.1]	25.3 [20.7, 30.4]
VIC	51.9 [47.2, 56.7]	24.8 [20.7, 29.2]	23.3 [19.6, 27.6]
QLD	53.0 [47.6, 58.3]	23.6 [19.3, 28.6]	23.3 [19.0, 28.3]
SA	47.4 [42.2, 52.6]	28.34 [23.80, 33.38]	24.27 [19.64, 29.59]
WA	63.0 [57.1, 68.5]	19.6 [15.3, 24.7]	17.4 [13.3, 22.5]
TAS	60.5 [55.4, 65.3]	24.7 [20.5, 29.5]	14.8 [11.6, 18.7]
ACT	62.5 [56.6, 68.1]	17.2 [13.0, 22.4]	20.3 [15.8, 25.5]
NT	54.9 [45.3, 64.1]	16.1 [10.8, 23.5]	29.0 [20.5, 39.2]

^*^Most disadvantaged = range 1–5.

^**^Medium range = range 6–8.

^***^Most advantaged = range 9–10.

^^^^Row % estimated presented.

^~^Total support = Strongly support + Somewhat support = Proportion in favour of the policy.

^@^Neutral = Proportion neither for nor against the policy.

^^^Total oppose = Strongly oppose + Somewhat oppose = Proportion against the policy.

^#^Less healthy food and beverage refer to discretionary food and drinks high in sugar, salt and saturated fats.

**Table 5:** Crosstabulation between demographic characteristics and support for prohibiting advertising and promotion of less healthy^#^ food and beverages to children under the age of 16 (weighted)^^^^

Demographic characteristics	Support for prohibiting advertising and promotion of less healthy food and beverages to children under the age of 16		
	Total support^~^ *n*	Neutral^@^ *n*	Total oppose^^^ *n*
Total sample (*n* = 3788)	2889	531	368
	% [95% confidence interval]		
	73.2 [71.1, 75.2]	15.4 [13.8, 17.2]	11.4 [9.9, 13.0]
Age (*n* = 4040) in years			
18–24	58.8 [48.1, 68.7]	26.5 [18.4, 36.5]	14.7 [8.5, 24.2]
25–44	75.6 [71.4, 79.3]	15.2 [12.2, 18.9]	9.2 [6.9, 12.2]
45–64	73.5 [69.9, 76.9]	14.5 [11.9, 17.6]	12.0 [9.6, 14.7]
65 and above	76.4 [73.3, 79.2]	13.0 [10.9, 15.5]	10.6 [8.5, 13.1]
Sex (*n* = 4040)			
Male	72.9 [69.7, 75.9]	16.1 [13.7, 18.9]	11.0 [9.0, 13.3]
Female	73.4 [70.6, 76.0]	14.8 [12.8, 17.1]	11.8 [9.9, 14.1]
Highest level of education attained (*n* = 3983)			
Tertiary	81.7 [78.5, 84.5]	10.4 [8.2, 13.2]	7.9 [6.0, 10.2]
Vocational	72.5 [69.2, 75.5]	15.6 [13.2, 18.3]	11.9 [9.8, 14.5]
Student	60.6 [48.3, 71.7]	21.9 [13.8, 32.8]	17.5 [9.6, 29.7]
Secondary	67.8 [62.9, 72.3]	19.4 [15.7, 23.6]	12.8 [9.9, 16.6]
Number of dependants (*n* = 4034)			
Households with children (0–14 years)	70.1 [64.9, 74.8]	16.8 [13.1, 21.2]	13.1 [9.7, 17.5]
Households with no children (>15 years)	73.9 [71.5, 76.01]	15.1 [13.4, 17.0]	11.0 [9.5, 12.8]
Household income (*n* = 3396)			
$100K and above	77.5 [73.2, 81.2]	12.2 [9.2, 16.0]	10.3 [7.9, 13.4]
50K to <100K	74.0 [69.6, 78.1]	16.4 [13.0, 20.5]	9.6 [7.1, 12.8]
<20K to <50K	72.7 [69.3, 75.8]	15.7 [13.3, 18.4]	11.6 [9.4, 14.4]
Area of residence (*n* = 4040)			
Most disadvantaged^*^	72.9 [69.8, 75.9]	16.6 [14.2, 19.2]	10.5 [8.6, 12.8]
Medium^**^	70.7 [66.4, 74.5]	15.7 [12.8, 19.2]	13.6 [10.7, 17.2]
Most advantaged^***^	76.2 [72.3, 79.6]	13.4 [10.7, 16.8]	10.4 [8.0, 13.3]
State of residence (*n* = 4040)			
NSW	72.2 [67.1, 76.9]	16.2 [12.4, 20.8]	11.6 [8.6, 15.3]
VIC	74.1 [69.7, 78.1]	13.9 [10.9, 17.7]	12.0 [9.1, 15.5]
QLD	73.0 [67.8, 77.6]	14.2 [10.8, 18.3]	12.8 [9.4, 17.3]
SA	70.7 [65.6, 75.3]	18.9 [15.2, 23.3]	10.4 [7.4, 14.3]
WA	73.6 [67.6, 78.7]	15.4 [11.4, 20.6]	11.0 [7.5, 15.9]
TAS	73.6 [68.7, 77.8]	18.6 [14.9, 23.1]	7.8 [5.4, 11.1]
ACT	81.2 [76.3, 85.3]	12.1 [8.8, 16.3]	6.7 [4.3, 10.3]
NT	71.0 [61.0, 79.3]	13.5 [8.3, 21.3]	15.5 [8.9, 25.5]

^*^Most disadvantaged = range 1–5.

^**^Medium range = range 6–8.

^***^Most advantaged = range 9–10.

^^^^Row % estimated presented.

^~^Total support = Strongly support + Somewhat support = Proportion in favour of the policy.

^@^Neutral = Proportion neither for nor against the policy.

^^^Total oppose = Strongly oppose + Somewhat oppose = Proportion against the policy.

^#^Less healthy food and beverage refer to discretionary food and drinks high in sugar, salt and saturated fats.

**Table 6:** Crosstabulation between demographic characteristics and support for restricting sugar-sweetened drinks from vending machines in all public buildings (weighted)^^^^

Demographic characteristics	Support for restricting sugar-sweetened drinks from vending machines in all public buildings		
	Total support^~^ *n*	Neutral^@^ *n*	Total oppose^^^ *n*
Total sample (*n* = 3783)	2304	840	639
	% [95% confidence interval]		
	58.0 [55.7, 60.2]	23.1 [21.3, 25.0]	19.0 [17.2, 20.80]
Age (*n* = 4040) in years			
18–24	47.0 [36.5, 57.8]	31.6 [22.9, 41.8]	21.4 [14.3, 30.8]
25–44	54.6 [50.2, 58.9]	22.8 [19.4, 26.7]	22.6 [18.9, 26.6]
45–64	58.5 [54.5, 62.2]	23.3 [20.3, 26.7]	18.2 [15.3, 21.6]
65 and above	63.7 [60.3, 67.0]	21.3 [18.6, 24.2]	15.0 [12.6, 17.8]
Sex (*n* = 4040)			
Male	56.8 [53.5, 60.2]	22.8 [20.1, 25.7]	20.4 [17.7, 23.3]
Female	59.0 [56.0, 61.8]	23.3 [20.9, 25.9]	17.7 [15.4, 20.2]
Highest level of education attained (*n* = 3983)			
Tertiary	64.7 [61.1, 68.2]	20.1 [17.3, 23.2]	15.2 [12.9, 17.9]
Vocational	58.5 [55.3, 68.1]	22.3 [19.4, 24.9]	19.2 [16.54, 22.3]
Student	46.7 [35.0, 58.9]	26.9 [18.0, 38.2]	26.4 [16.8, 38.6]
Secondary	51.2 [46.1, 56.3]	28.2 [24.0, 32.9]	20.6 [16.7, 25.07]
Number of dependants (*n* = 4034)			
Households with children (0–14 years)	51.5 [46.3, 56.6]	28.2 [23.8, 33.2]	20.3 [16.4, 24.8]
Households with no children (>15 years)	59.7 [57.2, 62.1]	21.7 [19.7, 23.7]	18.6 [16.7, 20.8]
Household income (*n* = 3396)			
$100K and above	60.2 [55.6, 64.5]	19.9 [16.5, 23.9]	19.9 [16.6, 23.8]
50K to <100K	56.5 [51.8, 61.0]	25.3 [21.4, 29.7]	18.2 [14.8, 22.1]
<20K to <50K	60.1 [56.5, 63.5]	22.9 [20.1, 26.0]	17.0 [14.4, 20.1]
Area of residence (*n* = 4040)			
Most disadvantaged^*^	57.4 [54.0, 60.8]	24.1 [21.4, 27.0]	18.5 [15.8, 21.5]
Medium^**^	57.0 [52.7, 61.1]	22.2 [19.0, 25.98]	20.8 [17.4, 24.6]
Most advantaged^***^	59.9 [55.9, 63.9]	22.5 [19.2, 26.2]	17.6 [14.7, 21.0]
State of residence (*n* = 4040)			
NSW	51.3 [45.9, 56.7]	28.2 [23.6, 33.3]	20.5 [16.2, 25.5]
VIC	59.6 [54.9, 64.1]	21.2 [17.7, 25.2]	19.2 [15.6, 23.5]
QLD	61.3 [55.9, 66.4]	19.7 [15.9, 24.3]	19.0 [15.0, 23.7]
SA	49.7 [44.4, 54.9]	28.7 [24.2, 33.6]	21.6 [17.3, 26.7]
WA	66.0 [60.2, 71.4]	19.0 [14.8, 24.2]	15.0 [11.3, 19.5]
TAS	60.7 [55.6, 65.6]	24.1 [19.9, 28.8]	15.2 [11.8, 19.4]
ACT	60.9 [54.9, 66.5]	20.7 [16.4, 25.9]	18.4 [14.0, 23.8]
NT	54.0 [44.5, 63.3]	23.4 [16.2, 32.7]	22.6 [15.1, 32.2]

^*^Most disadvantaged = range 1–5.

^**^Medium range = range 6–8.

^***^Most advantaged = range 9–10.

^^^^Row % estimated presented.

^~^Total support = Strongly support + Somewhat support = Proportion in favour of the policy.

^@^Neutral = Proportion neither for nor against the policy.

^^^Total oppose = Strongly oppose + Somewhat oppose = Proportion against the policy.

**Table 7:** Crosstabulation between demographic characteristics and support for restricting sugar-sweetened drinks from vending machines in schools (weighted)^^^^

Demographic characteristics	Support for restricting sugar-sweetened drinks from vending machines in schools		
	Total support^~^ *n*	Neutral^@^ *n*	Total oppose^^^ *n*
Total sample (*n* = 3788)	3026	433	329
	% [95% confidence interval]		
	77..4 [75.4, 79.3]	12.7 [11.2, 14.3]	9.9 [8.6, 11.5]
Age (*n* = 4040) in years			
18–24	66.8 [56.3, 75.9]	21.4 [14.1, 31.4]	11.8 [6.4, 20.3]
25–44	78.4 [74.4, 81.9]	11.8 [9.3, 15.0]	9.8 [7.2, 13.1]
45–64	79.0 [75.5, 82.0]	11.8 [9.4, 14.7]	9.2 [7.2, 11.89]
65 and above	79.4 [76.3, 82.1]	11.1 [9.1, 13.5]	9.5 [7.5, 11.9]
Sex (*n* = 4040)			
Male	76.8 [73.7, 79.5]	13.1 [11.0, 15.6]	10.1 [8.2, 12.5]
Female	77.9 [75.2, 80.4]	12.3 [10.4, 14.5]	9.8 [8.0, 12.0]
Highest level of education attained (*n* = 3983)			
Tertiary	84.5 [81.7, 87.0]	8.6 [6.8, 10.7]	6.9 [5.2, 9.1]
Vocational	77.4 [74.2, 80.2]	12.4 [10.2, 15.0]	10.2 [8.2, 12.7]
Student	64.4 [52.0, 75.0]	21.8 [13.6, 33.1]	13.8 [7.0, 25.4]
Secondary	72.6 [67.9, 76.9]	15.5 [12.2, 19.5]	11.9 [9.0, 15.6]
Number of dependants (*n* = 4034)			
Households with children (0–14 years)	74.7 [70.0, 79.1]	14.6 [11.3, 18.8]	10.7 [7.6, 14.8]
Households with no children (>15 years)	78.1 [75.9, 80.2]	12.1 [10.5, 13.8]	9.8 [8.4, 11.5]
Household income (*n* = 3396)			
$100K and above	83.9 [80.3, 87.0]	8.6 [6.3, 11.5]	7.5 [5.4, 10.3]
50K to <100K	78.2 [73.9, 81.9]	13.0 [10.0, 16.8]	8.8 [6.5, 11.7]
<20K to <50K	76.9 [73.6, 79.8]	13.0 [10.8, 15.7]	10.1 [8.1, 12.7]
Area of residence (*n* = 4040)			
Most disadvantaged^*^	75.7 [72.5, 78.6]	15.1 [12.7, 17.9]	9.2 [7.4, 11.5]
Medium^**^	78.0 [74.1, 81.4]	9.5 [7.3, 12.3]	12.5 [9.7, 15.9]
Most advantaged^***^	79.2 [75.5, 82.4]	12.5 [9.9, 15.6]	8.3 [6.2, 11.1]
State of residence (*n* = 4040)			
NSW	74.9 [69.7, 79.4]	14.6 [11.0, 19.1]	10.5 [7.6, 14.4]
VIC	75.8 [71.5, 79.9]	12.3 [9.5, 15.9]	11.9 [8.8, 15.4]
QLD	77.2 [72.3, 81.5]	12.5 [9.4, 16.4]	10.3 [7.3, 14.5]
SA	77.1 [72.4, 81.2]	13.0 [10.1, 16.7]	9.9 [7.00, 13.8]
WA	82.4 [77.0, 86.7]	10.9 [7.4, 15.7]	6.7 [4.2, 10.5]
TAS	79.7 [75.1, 83.6]	12.2 [9.1, 16.2]	8.1 [5.6, 11.5]
ACT	82.0 [77.1, 86.0]	10.6 [7.5, 14.8]	7.4 [4.9, 11.1]
NT	71.6 [61.8, 79.8]	16.5 [10.3, 25.3]	11.9 [6.5, 20.5]

^*^Most disadvantaged = range 1–5.

^**^Medium range = range 6–8.

^***^Most advantaged = range 9–10.

^^^^Row % estimated presented.

^~^Total support = Strongly support + Somewhat support = Proportion in favour of the policy.

^@^Neutral = Proportion neither for nor against the policy.

^^^Total oppose = Strongly oppose + Somewhat oppose = Proportion against the policy.

Younger adults aged 18–24 years expressed low level of support for all policy initiatives in comparison to older adults. However, both young and older adults expressed relatively strong support for policies targeting children. For example, more than 60% of the population across all age groups supported policies prohibiting advertising and promotion of less healthy food and beverages to children under the age of 16 and more than 67% supported policies limiting SSBs in vending machines in schools. More women than men expressed support for all policy initiatives. However, the difference observed between men and women was very small. An upward trajectory of support was observed for all policies across level of education. Participants with a tertiary level of education express high support for all policy initiatives and the support was exceptionally high (>80%) for policies prohibiting advertising and promotion of less healthy food and beverages to children under the age of 16 and for restricting sugar-sweetened drinks from vending machines in schools. Participants living with and without children expressed support for all policy initiatives. Overall, more than half of the participants living in the highest category of household income and in most socioeconomic advantage supported all policy initiatives to address unhealthy diet with some demonstrating a higher support (>70%) for policies prohibiting advertising and promotion of less healthy food and beverages to children under the age of 16 and for limiting SSBs from vending machines in schools than their counterparts. Similarly, three-fourth of residents across all states in Australia supported policies targeting children including policies prohibiting advertising and promotion of less healthy food and beverages and limiting SSBs from vending machines in schools.

## DISCUSSION

The findings of this study reveal that majority of the Australian population support policies to tackle unhealthy diet. The highest level of support was observed for policies targeting children while a relatively lower level of support was observed for polices proposing taxation, but even this was supported by over half of the respondents. The level of support for the various policies assessed was higher among the more affluent respondents and increased with age. Some differences in the level of support were observed between gender and none between participants living with or without children.

Our findings are in line with both national and international studies on the same topic. Similar to our study, previous studies from Australia and Canada have reported strong public support among adults for policy initiatives specifically for sugar-sweetened beverage (SSB) (Kongats *et al.*, 2019; Miller *et al.*, 2019; Richardson *et al.*, 2019). In our study, women, those with tertiary education and residing in most socioeconomic advantaged areas were more likely to support for policy initiatives to address unhealthy diet than their counterparts. Studies from the USA have similarly indicated that women are more likely than men to support public health initiatives or government policy (Kamas and Preston, 2019; Kock *et al.*, 2022) and tend to engage in and promote health-seeking behaviour (Manierre, 2015). Similar socio-demographic differences in support have also previously been observed in UK for tobacco and alcohol cessation strategies (Office for National Statistics, 2010). The public opinion on the policies reflects complex social and political context of the country that may contribute to the overall level of policy support. Similarly, although policies that restrict sales of SSBs, impose sugar tax or policies that target children are more likely to be supported by low free sugars consumers than their counterparts, there may be differential support among consumers of high free sugars themselves depending on their motivation to reduce. These factors lead to differences in support for policies addressing free sugars and must be investigated and addressed for successful implementation of policies addressing unhealthy diet at a population-level.

A variety of regulations targeting SSBs have been implemented in many countries but are still being contested in Australia. For example, in Latina America since 2006, 14 countries have adopted regulatory strategies to reduce SSB consumption including restrictions to SSB availability in schools, taxes, restrictions on advertising and marketing, labelling rules among others (Carriedo *et al.*, 2021). In Singapore, based on public consultation in 2019 (Ministry of Health Singapore, 2018), mandatory front-of-pack nutrition labels for less healthy pre-packaged SSBs and advertising prohibitions for the least healthy SSBs on local mass channels were introduced in 2021 (Ministry of Health Singapore, 2019). This indicates that while government leadership is critical, stronger cross-sectoral government leadership and commitment, enduring partnerships are also required to improve the social and environmental conditions to support healthier dietary choices. With no national nutritional policy recommendation for limiting free sugars intake, Australia lags behind more than 50 jurisdictions across the world on this matter.

’2 in 3 (67%) Australian adults have overweight or obesity. In 2018, overweight and obesity contributed to 8.4% of the total (fatal and non-fatal) burden of disease in Australia [i.e. 14.5 disability-adjusted life years (DALYs) per 1000 people] and 9.2 DALYs (per 1000 people) were attributed to dietary risks (Australian Institute of Health and Welfare, 2021a). It is thus clear that overweight and obesity is an ongoing challenge to the healthcare system. This study is timely as it highlights an urgent need to focus on advocacy efforts to raise awareness in the public to drive social movement to support population-level policies to tackle unhealthy diet. For example, similar to that in Canada (Alberta Policy Coalition for Chronic Disease Prevention, 2016; Coalition Poids, 2018), there are several health advocacy groups in Australia such as the Obesity Policy Coalition, the Public Health Association of Australia and others, that are dedicated to improving population health through strong advocacy. Australia’s commitment to WHO’s global target to halt the rise in overweight and obesity is an important opportunity for government, industry, the community, and individuals to work together to support healthy diet (World Health Organization, 2013). National Obesity Strategy 2022–2032 (Commonwealth of Australia, 2022) and the work of the Obesity Policy Coalition (Obesity Policy Coalition, 2018) can be used as a starting point to guide country’s actions to transform the overall food environment (physical and online) to support health and well-being. Collective and synergistic efforts are needed to create a social movement to drive political change to introduce healthy eating policies.

In the present study, ‘level of intrusiveness’ of policies addressing unhealthy diet may have influenced public support. Based on the Nuffield Intervention Ladder (Nuffield Council on Bioethics, 2007), the policies assessed in our study can be classified as low and high on intrusiveness depending on the target population for the intervention. Policies low on intrusiveness include those implemented in and around schools targeting children were more supported than policies high on intrusiveness including restricting choice in public places or taxation. A systematic review (Diepeveen *et al.*, 2013) showed that public support is often greatest for least intrusive initiatives to change health behaviours. Similar patterns in the support have also been observed among Canadian population (Kongats *et al.*, 2019). However, policies combining taxes on less healthy food (high on intrusiveness) with subsidies on healthier food (low on intrusiveness) garner increased public support due to multiple benefits of improving health and environment (Thow *et al.*, 2010; Black *et al.*, 2012; Ni Mhurchu *et al.*, 2013). Hence, it is essential and valuable to focus on both the importance of implementing multiple initiatives in harmony supporting each other and communicating evidence of multiple benefits of policies for increased support and trust on the Government’s commitment to health and well-being (Mantzari *et al.*, 2022). Australia is well positioned to introduce a comprehensive suite of policy initiatives to address unhealthy diet through strong regulatory framework and other prevention initiatives (Commonwealth of Australia, 2022). Australia has previously demonstrated a long-standing and successful leadership in tobacco control (Australian Institute of Health and Welfare, 2021b) through implementation of a regulatory framework and initiatives. This experience can help guide Australian government’s actions to tackle unhealthy diet.

### Policy and research implications

Public support for policy initiatives is critical for policymakers to achieve greater success with policy objectives. As a first step, making the process of seeking public opinion on multiple policies more transparent and open to public scrutiny may improve confidence among public that public health is a key priority for the government. This is important as often public opinions on public health policies are formed by competing messages received on the likely impact of initiatives from governments and commercial companies that may be misleading. Hence, government needs to step up to counteract disinformation through leveraging three critical components of any effective evidence-based public health policy, that is, transparency, communication and trust, as evident during COVID-19 pandemic (OECD, 2020; Martin *et al.*, 2022). Next, this study calls for better framing of policies (especially those that may be viewed by the public as unfavourable but are most effective, such as sugar tax) in ways that closely align intervention effectiveness with public acceptability (Barry *et al.*, 2013). Presenting multiple benefits of policies may potentially enable in enhancing public support (Wicki *et al.*, 2020; Mantzari *et al.*, 2022). Investigating determinants and stability of public preferences towards public health policies would be a useful next step. This would enable in designing and implementing initiatives that align with population’s dominant core values and beliefs, thus influencing acceptability.

### Strengths and limitations

A strength of this study is that a population-based survey data representing the Australian population was analysed to gather level of public support on public health polices through a pre-tested questionnaire (Kongats *et al.*, 2019). Next, as the survey questionnaire analysed in this study was adopted from Canada, the comparison between Australia and Canada made throughout offered meaningful insights. Some limitations of the study deserve acknowledgement. First, the questionnaire was nested in a nationwide oral health study and, therefore, the participants knew the causes of oral diseases, particularly the role of sugars in the development of dental caries. Consequently, desirability bias cannot be ruled out. Second, low response rate (27%) may have introduced non-response bias, hence the results presented may not be generalisable. Next, the reasons behind the lack of support, as reported in some international studies, was not investigated. Another important point to consider is that there may be some overlapping between the 95% confidence intervals and therefore it is necessary to perform the statistical test for the null hypothesis using the appropriate statistical analysis or compute the confidence interval for the difference in the parameters. Last, as this is a cross-sectional survey from 2018, our findings may not represent the most current status of policy support in Australia.

## CONCLUSION

The findings indicate that majority of the Australians support multiple policy initiatives addressing unhealthy diet with support ranging from 50 to 80% of the study population. Findings suggest that framing policies focussing on protecting children are likely to be more acceptable and have greater likelihood of achieving success.

## References

[CIT0001] Alberta Policy Coalition for Chronic Disease Prevention. (2016) Who we are. APCCP, Edmonton, Alberta.

[CIT0002] Australian Bureau of Statistics. (2016a) 2033.0.55.001—Census of Population and Housing: Socio-Economic Indexes for Areas (SEIFA). ABS, Canberra (AUST).

[CIT0003] Australian Bureau of Statistics. (2016b) 4364.0.55.011—Australian Health Survey: Consumption of Added Sugars [Press release].

[CIT0004] Australian Institute of Health and Welfare. (2021a) Australia’s Health 2020: Tobacco Smoking. AIHW, Canberra.

[CIT0005] Australian Institute of Health and Welfare. (2021b) Australian Burden of Disease Study: Impact and Causes of Illness and Death in Australia. AIHW, Australian Government, Canberra.

[CIT0006] Backholer, K., Blake, M. and Vandevijvere, S. (2017) Sugar-sweetened beverage taxation: an update on the year that was 2017. Public Health Nutrition, 20, 3219–3224, doi:10.1017/s1368980017003329. 29160766 PMC10261626

[CIT0007] Barry, C. L., Niederdeppe, J. and Gollust, S. E. (2013) Taxes on sugar-sweetened beverages: results from a 2011 national public opinion survey. American Journal of Preventive Medicine, 44, 158–163, doi:10.1016/j.amepre.2012.09.065. 23332333

[CIT0008] Bélanger-Gravel, A., Desroches, S., Janezic, I., Paquette, M. C. and De Wals, P. (2019) Pattern and correlates of public support for public health interventions to reduce the consumption of sugar-sweetened beverages. Public Health Nutrition, 22, 3270–3280, doi:10.1017/s1368980019002076. 31544722 PMC10260561

[CIT0009] Black, A. P., Brimblecombe, J., Eyles, H., Morris, P., Vally, H. and Dea, K. O. (2012) Food subsidy programs and the health and nutritional status of disadvantaged families in high income countries: a systematic review. BMC Public Health, 12, 1099, doi:10.1186/1471-2458-12-1099. 23256601 PMC3559269

[CIT0010] Carriedo, A., Koon, A. D., Encarnación, L. M., Lee, K., Smith, R. and Walls, H. (2021) The political economy of sugar-sweetened beverage taxation in Latin America: lessons from Mexico, Chile and Colombia. Globalization and Health, 17, 5, doi:10.1186/s12992-020-00656-2. 33402166 PMC7786456

[CIT0011] Chrisopoulos, S., Ellershaw, A., Do, L. and Luzzi, L. (2019) Chapter 2: Study aims and methods. In ARCPOH. Australia’s Oral Health: National Study of Adult Oral Health 2017–18. The University of Adelaide, South Australia, Adelaide.

[CIT0012] Coalition Poids. (2018) Homepage. http://www.cqpp.qc.ca/en/ (accessed May 2022).

[CIT0013] Colchero, M. A., Popkin, B. M., Rivera, J. A. and Ng, S. W. (2016) Beverage purchases from stores in Mexico under the excise tax on sugar sweetened beverages: observational study. BMJ, 352, h6704, doi:10.1136/bmj.h6704. 26738745 PMC4986313

[CIT0014] Commonwealth of Australia. (2022). The National Obesity Strategy 2022-2032. Health Ministers Meeting. Updated March 18, 2022. https://www.health.gov.au/resources/publications/national-obesity-strategy-2022-2032. Accessed May 2022.

[CIT0015] Diepeveen, S., Ling, T., Suhrcke, M., Roland, M. and Marteau, T. M. (2013) Public acceptability of government intervention to change health-related behaviours: a systematic review and narrative synthesis. BMC Public Health, 13, 756, doi:10.1186/1471-2458-13-756. 23947336 PMC3765153

[CIT0016] Donaldson, E., Cohen, J., Rutkow, L., Villanti, A., Kanarek, N. and Barry, C. (2015) Public support for a sugar-sweetened beverage tax and pro-tax messages in a Mid-Atlantic US state. Public Health Nutrition, 18, 2263–2273. 25430945 10.1017/S1368980014002699 PMC4447609

[CIT0017] Ellershaw, A., Chrisopoulos, S. and Luzzi, L. (2020) National Study of Adult Oral Health 2017–18: participation and representativeness. Australian Dental Journal, 65, S11–S17, doi:10.1111/adj.12759. 32583590

[CIT0018] Global Food Research Program. (2020) Sugary Drink Taxes Around the World. University of North Carolina at Chapel Hill, Chapel Hill.

[CIT0019] Gollust, S. E., Barry, C. L. and Niederdeppe, J. (2014) Americans’ opinions about policies to reduce consumption of sugar-sweetened beverages. Preventive Medicine, 63, 52–57, doi:10.1016/j.ypmed.2014.03.002. 24631499

[CIT0020] Gupta, A., Miller, C., Harford, J., Smithers, L. and Braunack-Mayer, A. (2019) Australia’s sugar tale. Public Health Nutrition, 22, 2682–2687. 31120007 10.1017/S1368980019001228 PMC10277187

[CIT0021] Gupta, A., Smithers, L., Braunack-Mayer, A. and Harford, J. (2018a) How much free sugar do Australians consume? Findings from a national survey. Australian and New Zealand Journal of Public Health, 42, 533–540. 30296823 10.1111/1753-6405.12836

[CIT0022] Gupta, A., Smithers, L., Harford, J., Merlin, T. and Braunack-Mayer, A. (2018b) Determinants of knowledge and attitudes about sugar and the association of knowledge and attitudes with sugar intake among adults: a systematic review. Appetite, 1, 185–194. 10.1016/j.appet.2018.03.019 29634988

[CIT0023] Kamas, L. and Preston, A. (2019) Can empathy explain gender differences in economic policy views in the United States? Feminist Economics, 25, 58–89.

[CIT0024] Kock, L., Shahab, L., Moore, G., Shortt, N. K., Pearce, J. and Brown, J. (2022) Assessing the profile of support for potential tobacco control policies targeting availability in Great Britain: a cross-sectional population survey. Tobacco Control, doi:10.1136/tc-2022-057508. 36008128

[CIT0025] Kongats, K., McGetrick, J., Raine, K., Voyer, C. and Nykiforuk, C. (2019) Assessing general public and policy influencer support for healthy public policies to promote healthy eating at the population level in two Canadian provinces. Public Health Nutrition, 22, 1492–1502. 30782230 10.1017/S1368980018004068 PMC10260847

[CIT0026] Kwon, J., Cameron, A. J., Hammond, D., White, C. M., Vanderlee, L., Bhawra, J. et al. (2019) A multi-country survey of public support for food policies to promote healthy diets: findings from the International Food Policy Study. BMC Public Health, 19, 1205, doi:10.1186/s12889-019-7483-9. 31477071 PMC6721115

[CIT0027] Manierre, M. J. (2015) Gaps in knowledge: tracking and explaining gender differences in health information seeking. Social Science and Medicine, 128, 151–158, doi:10.1016/j.socscimed.2015.01.028. 25618604

[CIT0028] Mantzari, E., Reynolds, J. P., Jebb, S. A., Hollands, G. J., Pilling, M. A. and Marteau, T. M. (2022) Public support for policies to improve population and planetary health: a population-based online experiment assessing impact of communicating evidence of multiple versus single benefits. Social Science and Medicine, 296, 114726, doi:10.1016/j.socscimed.2022.114726. 35093794 PMC8907862

[CIT0029] Martin, A., Mikołajczak, G., Baekkeskov, E. and Hartley, K. (2022) Political stability, trust and support for public policies: a survey experiment examining source effects for COVID-19 interventions in Australia and Hong Kong. International Journal of Public Opinion Research, 34(3), edac024. doi:10.1093/ijpor/edac024.

[CIT0030] Miller, C. L., Dono, J., Wakefield, M. A., Pettigrew, S., Coveney, J., Roder, D. et al. (2019) Are Australians ready for warning labels, marketing bans and sugary drink taxes? Two cross-sectional surveys measuring support for policy responses to sugar-sweetened beverages. BMJ Open, 9, e027962, doi:10.1136/bmjopen-2018-027962. PMC6597645 31248926

[CIT0031] Ministry of Health Singapore. (2018) Public Consultation on measures to reduce sugar intake from pre-packaged sugar-sweetened beverages [Press release]

[CIT0032] Ministry of Health Singapore. (2019) To introduce measures to reduce sugar intake from pre-packaged sugar-sweetened beverages [Press release]

[CIT0033] Mozaffarian, D., Angell, S., Lang, T. and Rivera, J. (2018) Role of government policy in nutrition-barriers to and opportunities for healthier eating. BMJ, 361, k2426. 29898890 10.1136/bmj.k2426 PMC5997034

[CIT0034] Ni Mhurchu, C., Eyles, H., Schilling, C., Yang, Q., Kaye-Blake, W., Genç, M. et al. (2013) Food prices and consumer demand: differences across income levels and ethnic groups. PLoS One, 8, e75934, doi:10.1371/journal.pone.0075934. 24098408 PMC3788811

[CIT0035] Nuffield Council on Bioethics. (2007) Nuffield council on bioethics: policy process and practice. In Public Health: Ethical Issues. Nuffield Council on Bioethics, London.

[CIT0036] Nykiforuk, C. I. J., McGetrick, J. A., Raine, K. D. and Wild, T. C. (2019) Advocacy coalition impacts on healthy public policy-oriented learning in Alberta, Canada (2009–2016): a difference-in-differences analysis. Social Science and Medicine, 220, 31–40, doi:10.1016/j.socscimed.2018.10.017. 30391639

[CIT0037] Obesity Policy Coalition. (2018) Policy Brief: A Comprehensive Policy Program to Reduce Consumption of Sugar-Sweetened Beverages in Australia. Obesity Policy Coalition, Melbourne, Australia.

[CIT0038] OECD. (2020) Transparency, communication and trust: the role of public communication in responding to the wave of disinformation about the new Coronavirus. OECD Policy Responses to Coronavirus (COVID-19), OECD Publishing, Paris, 10.1787/bef7ad6e-en.

[CIT0039] Office for National Statistics. (2010) Population Trends, 141th edition. In Smith, C. (ed.). Office for National Statistics, UK.

[CIT0040] Peres, M. and Brennan, D. (2020) Oral health of Australian adults: achievements and challenges. Australian Dental Journal, Special Supplement.

[CIT0041] PLACE Research Lab. (2015) *Chronic Disease Prevention Survey*. http://placeresearchlab.com/Chronic-Disease-Prevention-Survey/, (accessed May 2022).

[CIT0042] Richardson, T., Yanada, B., Watters, D., Stupart, D., Lamichhane, P. and Bell, C. (2019) What young Australians think about a tax on sugar-sweetened beverages. Australian and New Zealand Journal of Public Health, 43, 63–67. 30548948 10.1111/1753-6405.12858

[CIT0043] Rivard, C., Smith, D., McCann, S. E. and Hyland, A. (2012) Taxing sugar-sweetened beverages: a survey of knowledge, attitudes and behaviours. Public Health Nutrition, 15, 1355–1361, doi:10.1017/s1368980011002898. 22269063 PMC4778078

[CIT0044] Sacks G. ; For the Food-EPI Australia project team. (2017) Policies for Tackling Obesity and Creating Healthier Food Environments: Scorecard and Priority Recommendations for the Australian Federal Government. Deakin University, Melbourne.

[CIT0045] Silver, L. D., Ng, S. W., Ryan-Ibarra, S., Taillie, L. S., Induni, M., Miles, D. R. et al. (2017) Changes in prices, sales, consumer spending, and beverage consumption one year after a tax on sugar-sweetened beverages in Berkeley, California, US: A before-and-after study. PLoS Medicine, 14, e1002283, doi:10.1371/journal.pmed.1002283. 28419108 PMC5395172

[CIT0046] Thow, A. M., Jan, S., Leeder, S. and Swinburn, B. (2010) The effect of fiscal policy on diet, obesity and chronic disease: a systematic review. Bulletin of the World Health Organization, 88, 609–614, doi:10.2471/blt.09.070987. 20680126 PMC2908970

[CIT0047] Wicki, M., Huber, R. A. and Bernauer, T. (2020) Can policy-packaging increase public support for costly policies? Insights from a choice experiment on policies against vehicle emissions. Journal of Public Policy, 40, 599–625, doi:10.1017/S0143814X19000205.

[CIT0048] World Health Organization. (2003) Diet, nutrition and the prevention of chronic diseases: report of a Joint WHO/FAO Expert Consultation WHO Technical Report Series, No. 916. World Health Organization, Geneva. 12768890

[CIT0049] World Health Organization. (2013) Global action plan for the prevention and control of non-communicable diseases 2013–2020. Geneva. http://apps.who.int/iris/bitstream/10665/94384/1/9789241506236_eng.pdf?ua=1 (accessed May 2022).

[CIT0050] World Health Organization. (2022) The Global NCD Compact 2020–2030.

